# Systemic inflammation accelerates tau propagation in an experimental model of Alzheimer’s disease

**DOI:** 10.1002/alz.089767

**Published:** 2025-01-03

**Authors:** Sarah Howard, Zeshan Ahmed, Katrin Deinhardt, Jessica Teeling

**Affiliations:** ^1^ University of Southampton, Southampton, na United Kingdom; ^2^ Eli Lilly & Co Ltd, Windlesham United Kingdom; ^3^ University of Southampton, Southampton United Kingdom

## Abstract

**Background:**

Systemic inflammation in patients with Alzheimer’s disease (AD) has been associated with an exacerbation in cognitive decline, but the underlying mechanisms remain largely unknown. In AD, intraneuronal hyperphosphorylated tau spreads through the brain via trans‐synaptic prion‐like propagation. Evidence suggests that propagation of tau pathology is linked to neuroinflammation. Here we aim to investigate if a low‐grade systemic bacterial infection drives neuroinflammation and exacerbates the spread of tau using an experimental model of AD

**Method:**

C57BL/6 mice underwent intracerebral injection of vehicle or AT8‐positive human tau lysate, isolated from post‐mortem AD tissue. Two months post‐injection, mice were given a systemic injection of saline or a life bacterial strain. Body and spleen weight was measured to confirm infection and brain tissue was collected 4 weeks post‐infection to assess tau pathology and markers of neuroinflammation.

**Result:**

We observed tau pathology in mice exposed to human tau lysate. Pathology in the dorsal fornix and the supramammillary nuclei was observed only in mice exposed to a bacterial infection. We also examined the inflammatory markers MHCII and CD64 and detected increased expression in mice exposed to a bacterial infection. The inflammatory response was localised to regions where pathology was observed, such as the CA3‐dentate gyrus axis.

**Conclusion:**

Following intracerebral injection of human tau lysate, propagation of tau was induced both dorsal and ventral to the injection site. The extend of pathological tau was exaggerated by the presence of low‐grade systemic inflammation. This suggests that systemic inflammatory episodes in AD patients may induce greater propagation of tau pathology.

